# Epithelial–mesenchymal transition in cutaneous fibrosis disease: from mechanisms to therapy

**DOI:** 10.3389/fimmu.2026.1834346

**Published:** 2026-06-09

**Authors:** ShaoXiang Yuan, Ziyi Luo, Nina Yang, Tao Xiong, YueZhong Chen, Xichao Jian, Yun Wang, Shune Xiao, Junzhe Chen, Chengliang Deng

**Affiliations:** 1 Department of Burns and Plastic Surgery, Affiliated Hospital of Zunyi Medical University, Zunyi Guizhou, China; 2 Collaborative Innovation Center of Tissue Repair and Regenerative Medicine, Zunyi Guizhou, China

**Keywords:** chronic inflammation, cutaneous fibrosis, epigenetic, epithelial–mesenchymal transition, fibroblasts, therapy

## Abstract

Cutaneous fibrosis takes place due to chronic inflammation, tissue trauma, or autoimmune reactions. It is marked by abnormal tissue remodeling; such remodeling is characterized by a raised quantity of myofibroblasts and an excessive accumulation of extracellular matrix (ECM) in the dermis. Therefore, this abnormal process brings about a decrease in tissue function, causes structural irregularities, and thus severely lowers patients’ quality of life. Although remarkable progressions have been achieved in preventive and treatment methods, the molecular processes under cutaneous fibrosis are not fully comprehended. Epithelial–mesenchymal transition (EMT) is a dynamic and strictly controlled biological phenomenon where epithelial cells discard their epithelial characteristics and obtain mesenchymal properties. Hence, newly appearing data has demonstrated that EMT plays a critical role in the development of fibrotic disorders. EMT has been proposed as a potential contributor to fibrogenesis, although its quantitative contribution and pathological significance relative to resident fibroblasts and other mesenchymal cell sources remain to be further clarified. These findings not only increase our knowledge of the pathophysiology of cutaneous fibrosis but also identify new molecular targets that can be utilized for therapeutic aims. This review collects the latest progressions in understanding the role of EMT in cutaneous fibrotic disorders. The objective is to provide new understandings into the regulatory mechanisms and possible treatment ways for this condition.

## Introduction

The skin, the largest organ in humans, covers nearly the whole body and contains a highly specialized immune microenvironment that is critical for maintaining tissue homeostasis, host defence, and wound repair (1). Under physiological conditions, tissue homeostasis of the dermal extracellular matrix (ECM) requires precise control of matrix deposition and remodeling. However, this equilibrium is perturbed in the context of chronic inflammation, persistent tissue injury, or autoimmune dysregulation, resulting in excessive ECM accumulation within the dermis and the onset of cutaneous fibrotic disorders (2–4). As fibrosis progresses, the skin becomes progressively thickened, rigid, and less elastic, culminating in contractures and restricted joint mobility (5). These pathological alterations severely compromise both dermal architecture and function, leading to substantial impairments in patients’ quality of life. Despite recent advances in elucidating the molecular and cellular mechanisms underlying fibrosis, current therapeutic strategies for cutaneous fibrosis remain largely inadequate for achieving meaningful clinical outcomes.

Cutaneous fibrotic disorders feature sustained immune activation with progressive fibrogenesis. During the initial inflammatory phase, infiltrating immune cells contribute to host defence, facilitate tissue clearance, and initiate reparative programs. As fibrosis progresses, sustained inflammation promotes the continuous release of profibrotic mediators, driving fibroblast activation and differentiation into myofibroblasts, which constitute a principal cellular source of ECM deposition (6); however, tissue-resident fibroblasts and myofibroblasts have traditionally been regarded as the dominant contributors to fibrotic tissue, and accumulating evidence indicates that EMT (Epithelial–Mesenchymal Transition) may also act as an immediate cellular origin of fibroblasts and myofibroblasts in fibrogenesis (7).

EMT is a dynamic biological process in which epithelial cells acquire mesenchymal characteristics. In this process, epithelial cells gradually lose their epithelial characteristics and functions and acquire mesenchymal-like attributes (8). Alongside its reversible counterpart, mesenchymal-epithelial transition (MET), EMT occurs in a wide range of situations, from normal developmental processes to pathological conditions. Scholars have proposed three distinct subtypes of EMT, each associated with specific biological environments. Type I EMT occurs during embryogenesis and organogenesis. For example, it takes place during the migration of neural crest cells and the development of cardiac valves. This subtype is crucial for the generation of primary mesenchymal cells, which can then undergo MET to form primary epithelial cell lineages (9). In cancer, malignant cells utilize Type III EMT to gain invasive abilities. These abilities enable the cells to metastasize and develop treatment resistance, which are definitive features of cancer progression to a malignant state (10). Type II EMT is mainly associated with tissue repair and organ fibrosis. In this subtype, continuous inflammation or tissue damage stimulates epithelial cells to adopt a myofibroblast-like form. This phenotypic change contributes to the reconstruction of tissues damaged by wounds or chronic injury (11). However, when excessive or prolonged, EMT promotes pathological ECM accumulation and drives progressive tissue fibrosis. Growing evidence suggests that EMT also contributes substantially to multiple types of cutaneous fibrosis. The skin consists of the epidermis, basement membrane, dermis, and subcutaneous tissue, the epidermal basal layer is anchored to the papillary dermis through the basement membrane. Under fibrotic conditions, basal keratinocytes may undergo EMT, penetrate the basement membrane, and migrate into the papillary dermis, thereby contributing to dermal fibrogenesis and pathological remodelling (12).

Taken together, these findings indicate that EMT targeting may offer a promising therapeutic approach for treating cutaneous fibrosis. This review integrates recent findings that define the contribution of EMT to cutaneous fibrotic disease, aiming to provide a conceptual framework and mechanistic insights that could guide the development of novel therapeutic interventions.

## EMT

**Figure 1:**
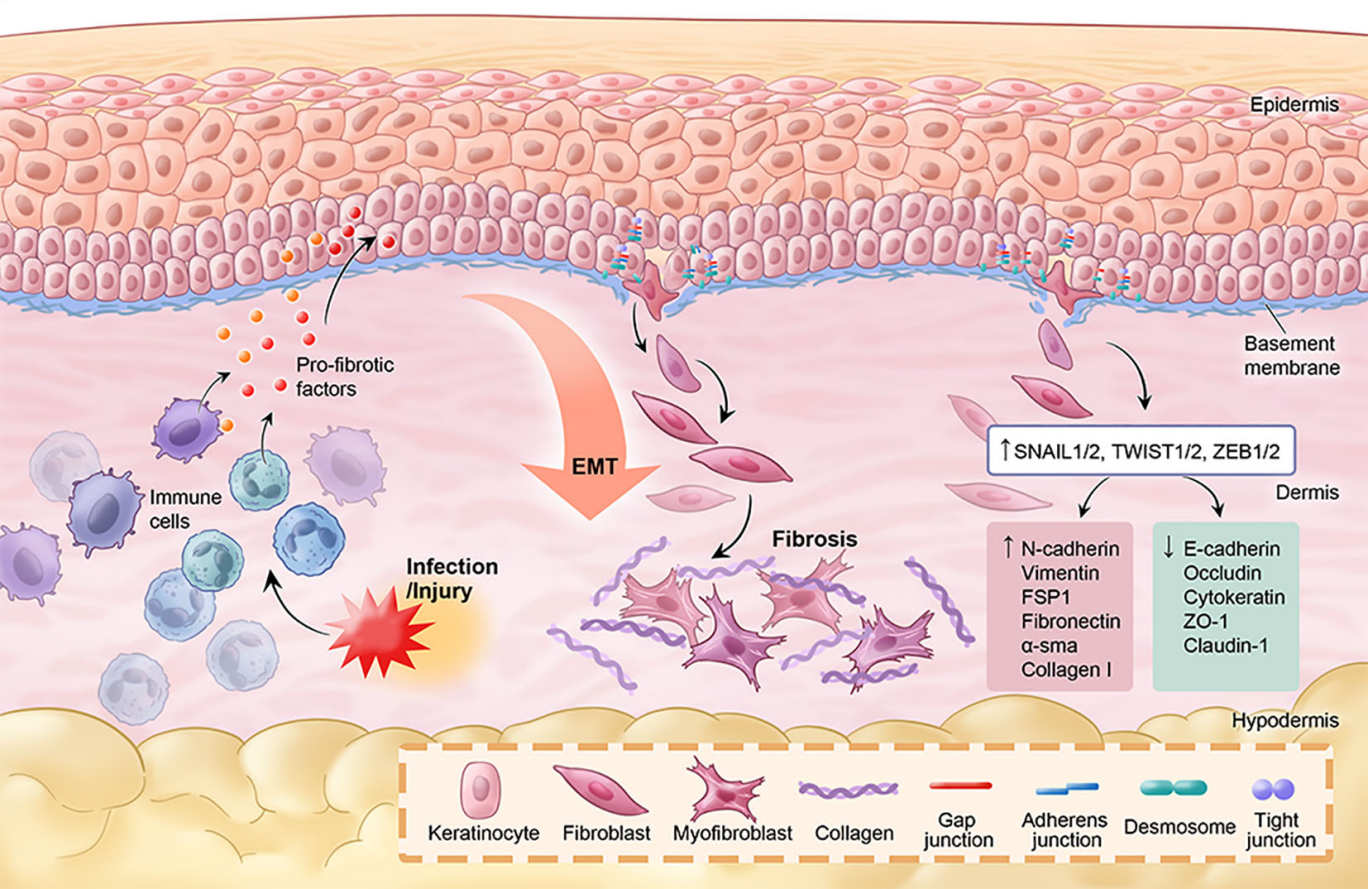
Basal keratinocyte EMT drives dermal fibrogenesis in cutaneous fibrosis. During chronic inflammation triggered by injury or infection, elevated levels of profibrotic factors released by immune cells induce EMT in basal keratinocytes. This process involves the dissolution of intercellular junctions (including tight junctions, adherens junctions, anchoring junctions, and gap junctions), loss of AB polarity, and cytoskeletal remodelling. As a result, keratinocytes adopt a mesenchymal phenotype marked by elevated levels of mesenchymal markers and EMT-TFs, along with downregulation of epithelial markers. These EMT-like cells migrate into the dermis and may promote dermal fibrosis by enhancing epithelial plasticity, fibroblast activation, and ECM deposition.

EMT is a highly orchestrated and multifaceted biological process. A variety of molecular cues derived from the ECM and the surrounding microenvironment engage specific receptors on the epithelial cell surface, triggering the activation of multiple intracellular signalling cascades. These pathways converge in a coordinated fashion and are integrated at the transcriptional level, collectively driving the EMT program, which is characterized by the suppression of epithelial gene expression and the induction of mesenchymal-associated transcriptional networks. The key morphological and phenotypic changes of basal keratinocytes during EMT in cutaneous fibrosis are summarized in **Figure 1**.

### Morphological transformation of epithelial cells during EMT

As EMT proceeds, epithelial cells progressively dismantle intercellular junctions and apical–basal (AB) polarity, accompanied by extensive cytoskeletal reorganization. These coordinated events culminate in pronounced morphological changes and together with a shift toward mesenchymal-like properties, characterized by enhanced migration and invasion, as well as the capacity to synthesize and secrete ECM components (13).

Intercellular junctions represent a fundamental structural foundation for preserving epithelial integrity and are broadly classified into tight junctions, adherens junctions, anchoring junctions, and gap junctions (14). Tight junctions, one of these junctional complexes, are positioned at the apical region of epithelial cells and are formed by transmembrane proteins such as claudins and occludins in association with cytoplasmic scaffold proteins, including zonula occludens (ZO) family members (15). These structures seal the intercellular space, thereby maintaining epithelial barrier function and AB polarity. Adherens junctions mediate intercellular cohesion within epithelial layers and are governed primarily by the transmembrane protein E-cadherin, which connects to the actin cytoskeleton via β-catenin, α-catenin, and p120-catenin (16). Anchoring junctions provide mechanical stability by connecting intermediate filaments to either neighbouring cells or the basement membrane and are composed of desmosomal cadherins, including desmogleins, desmocollins, plakoglobins, and plakophilins (17). Gap junctions establish direct cytoplasmic continuity between adjacent cells via intercellular channels formed by connexons, which are hexameric assemblies of connexin proteins. These channels enable the passage of small molecules and ions (18).

Upon EMT activation, intercellular junctions are destabilized and progressively disassembled. The level of tight junction proteins, such as claudin-1, occludins, and ZO-1, is markedly downregulated. Concurrently, the epithelial transmembrane protein E-cadherin is replaced by N-cadherin, leading to weaker and more dynamic intercellular adhesions (19). In addition, anchoring junctions and gap junctions undergo protein degradation or structural remodelling, further compromising epithelial integrity during EMT (20).

Apical-basal polarity is fundamental to epithelial barrier integrity and vectorial transport during development and tissue homeostasis. This polarity program governs the ordered assembly of tight junctions, adherens junctions, and desmosomes, while the Par, Crumbs, and Scribble complexes demarcate the apical and basolateral membrane domains (21, 22). Epithelial cells are further anchored to the underlying basement membrane through integrin-containing hemidesmosomes, which couple extracellular matrix adhesion to the intracellular cytokeratin network and thereby reinforce epithelial architecture. During EMT, however, cells must disengage from this highly organized epithelial state to acquire migratory competence. This transition requires disruption of apical-basal polarity, attenuation of cell-cell and cell-basement membrane adhesion, and establishment of front-rear polarity (23). Consequently, the loss of polarity compromises the spatial organization of junctional proteins and cytoskeletal support, destabilizing tight and adherens junctions and weakening intercellular adhesion. In contrast, mesenchymal cells lack functional epithelial junctions and are characterized by actin stress fibers organized along a front-rear axis. In parallel, the cytoskeleton undergoes extensive remodelling. The intermediate filament network transitions from cytokeratin-based structures, which provide mechanical stability, to a more flexible, vimentin-based framework (24); simultaneously, actin filament polymerization is progressively enhanced (19, 25). These changes collectively drive a marked morphological transformation, as epithelial cells shift from a cuboidal or columnar shape to an elongated, spindle-like phenotype, accompanied by the formation of lamellipodia and filopodia. During EMT, epithelial cells may secrete matrix metalloproteinases (MMPs), which remodel the ECM and disrupt basement membrane integrity (26, 27). By degrading ECM and basement membrane components, such as collagens, fibronectin, and other basement membrane components, MMPs weaken cell–cell and cell–matrix adhesion, facilitating epithelial detachment, mesenchymal-like transition, and subsequent migration or invasion into the dermis (28). MMP-2 and MMP-9 are among the most frequently implicated enzymes, mainly owing to their capacity to degrade type IV collagen and other basement membrane constituents (29).

### Core EMT transcription factors

EMT is tightly governed at the transcriptional level, which is crucial for shaping epithelial lineage plasticity during fibrogenesis (30, 31). Central to this process is a set of core transcription factors (TFs) that coordinate gene regulatory programs associated with EMT. These include members of the SNAIL family of transcriptional repressors, including SNAI1 and SNAI2 (also known as Slug) (32); the TWIST family of basic helix–loop–helix (bHLH) transcription factors, including TWIST1 and TWIST2 (33); and the zinc-finger E-box-binding (ZEB) transcription factors, consisting of ZEB1 and ZEB2, which also play essential roles in EMT induction (34).

#### SNAIL family members

SNAI1 and SNAI2 are zinc finger transcription factors of the SNAIL family. Both factors associate with the E-cadherin promoter and markedly suppress transcription (35). In addition, SNAI1 downregulates a range of epithelial markers, particularly tight junction proteins, such as claudin and occludin (36), and SNAI1 has been shown to cooperate with TWIST1 to promote ZEB1 expression, thereby reinforcing the EMT-associated transcriptional network (37). Furthermore, increasing evidence has indicated that SNAI1 can induce the expression of MMPs, thereby linking EMT-related transcriptional reprogramming to ECM remodelling (38).

#### ZEB family members

ZEB1 and ZEB2 are zinc finger transcription factors belonging to the ZEB family. Like SNAI1, ZEB1 binds to the E-cadherin promoter, resulting in strong transcriptional silencing (39). Furthermore, ZEB1 promotes the expression of mesenchymal-associated genes, including vimentin and N-cadherin, by recruiting transcriptional coactivator complexes to their respective promoters (40).

#### TWIST family members

TWIST1 and TWIST2 function as bHLH transcriptional regulators. Like SNAI1 and ZEB1, TWIST1 represses the level of the epithelial marker gene CDH1 by recruiting transcriptional corepressor complexes to its promoter while simultaneously activating mesenchymal marker genes such as N-cadherin (41). Notably, Hypoxia, or forced expression of hypoxia-inducible factor 1α (HIF-1α), has been demonstrated to drive EMT through TWIST induction. Consistent with this, HIF-1α associates with hypoxia-response elements located in the proximal TWIST promoter, thereby regulating its transcriptional activity (42).

The EMT-TFs SNAIL, ZEB, and TWIST exhibit distinct functional tendencies during EMT, including the suppression of epithelial gene program, the regulation of phenotypic plasticity, and the activation of migration-associated transcriptional programs. However, the roles of these core EMT-TFs are not strictly compartmentalized; instead, they frequently overlap in pathological settings. These TFs often function cooperatively and converge on common signalling pathways to coordinate regulation of the initiation and progression of EMT. It should also be noted that EMT-TFs do not necessarily exert a uniformly pro-fibrotic role across all fibrotic contexts. Evidence from fibrosis models in other organs suggests that genetic perturbation of certain EMT-TFs does not consistently produce antifibrotic effects and may, in specific cellular settings, even aggravate fibrotic phenotypes (43, 44). Collectively, these observations indicate that EMT-associated transcriptional programs are highly context-dependent and that fibrotic progression may be driven by multiple interconnected and compensatory pathogenic pathways.

### Molecular signaling networks governing EMT

EMT is best understood not as the consequence of a single linear pathway, but as a coordinated cell-state transition driven by the convergence of multiple signaling networks. Representative signalling pathways and therapeutic targets associated with EMT in cutaneous fibrotic diseases are summarized in **Table 1**.

**Table 1:** Signalling pathways and therapeutic targets associated with EMT in cutaneous fibrotic diseases.

Disease		Factor	Pathway	Result	Inhibitor
Pathologic scars	Keloids	\	TGF-β	TGF-β1 induces EMT in keloid-derived keratinocytes through SMAD2/SMAD3 signalling and ERK1/2 signalling (83).	SB525334/U0126
		\	TGF-β	TGF-β1/SMAD3 signalling induces EMT in keloid-derived keratinocytes (70).	\
		HIPK2	TGF-β	HIPK2 induces EMT in normal keratinocytes through TGF-β1/SMAD3 signalling (71).	\
		\	TGF-β	Pirfenidone inhibits TGF-β1-mediated EMT in keratinocytes from healthy skin as well as keloid tissue (81).	Pirfenidone
		\	TGF-β	Kelulut honey inhibits TGF-β-mediated EMT (82).	Kelulut honey
		PDE4B	TGF-β	Roflumilast inhibits PDE4B, thereby suppressing TGF-β1-induced phosphorylation of Smad3 and ERK1/2 and inhibiting EMT (73).	Roflumilast
		HIF - 1 α	\	A hypoxic microenvironment with increased HIF-1α induces EMT in keloid-derived keratinocytes (58).	\
		HIF-1α/PKM2	HIF-1	Metformin eliminates hypoxia-induced EMT in keloid-derived keratinocytes by inhibiting HIF-1α/PKM2 signalling (74).	Metformin
		\	PTEN/PI3K/AKT	β-sitosterol suppresses EMT in keloids by modulating the PTEN/PI3K/AKT signalling pathway (78).	β-sitosterol
		ERG	Wnt/β-catenin	ERG transcriptionally activates SFRP1 through the Wnt3a/β-catenin pathway, thereby promoting suppressing EMT (77).	\
		IL-6	JAK/STAT	IL-6 released from Wnt5A-stimulated keloid fibroblasts triggers JAK/STAT signalling in keratinocytes, thereby promoting EMT (75)..	\
		HIF-1α	MAPK/ERK	SCH772984 inhibits ERK1/2, thereby suppressing HIF-1α activation and inhibiting EMT (80).	SCH772984
		CYP24A1	Vitamin D	Treatment with vitamin D or the CYP24A1 activity inhibitor VID400 reverses EMT in keloid-derived keratinocytes (76).	VID400/Vitamin D
		\	\	In a study of 18 patients with keloids, nine patients received HBOT, which improved the hypoxic microenvironment and reduced the expression of EMT markers in keloid tissues (85).	HBOT
		\	\	Ribavirin inhibits EMT and may serve as a candidate therapeutic agent for scar treatment (86).	Ribavirin
		\	\	The findings support the involvement of EMT in keloid progression (69).	\
	HSs	TGF-β1	PTEN/PI3K/AKT	BTXA inhibits TGF-β1-induced PTEN methylation and reduces phosphorylation of PI3K and Akt, thereby attenuating EMT (99).	BTXA
		Exosomes	SMAD/TAK1	Fibroblast-derived exosomes induce EMT by activating the SMAD and TAK1 signalling pathways (96).	\
		Slit1	SMAD-dependent/SMAD-independent	Slit1 promotes EMT by upregulating SMAD and non-SMAD signalling pathways (94).	\
		PU.1	\	The transcription factor PU.1 promotes EMT in keratinocytes by directly binding to promoter regions and transcriptionally activating S100A8 and S100A9 expression (97).	\
		Caveolin1	Wnt/β-catenin	Downregulation of caveolin-1 increases β-catenin and TCF/LEF-1 transcriptional activity, thereby enhancing dehydration-induced EMT (93).	\
		\	\	In HSs, the epidermis is markedly thickened, and epidermal cells adjacent to the basement membrane express EMT markers (87).	\
		\	\	The downregulation of EMT markers following ESWT treatment in HSs suggests that inhibition of EMT may underlie its antifibrotic effects (98)..	ESWT
		\	\	Hydroxypyridone antifungal agents effectively inhibit EMT in keratinocytes (100).	hydroxypyridone
Autoimmune diseases	SSc	\	TGF-β	Increased levels of phosphorylated SMAD2 and SMAD3 indicate that TGF-β and SMAD signalling contributes to EMT in SSc (103).	\
		\	TGF-β	Nimbolide markedly inhibits TGF-β and Smad signalling and suppresses the EMT process triggered by this pathway (111).	Nimbolide
		Brachyury	TGF-β	Silencing Brachyury reduces type I collagen expression in fibroblasts derived from healthy controls and patients with SSc (104).	\
		\	TGF-β	LG283 inhibits EMT by suppressing the TGF-β- Smad-Snail axis (110).	LG283
		\	TGF-β	Aberrant amplification of the TGF-β–Ras–RAF–ERK and RalGDS pathways may enhance EMT in SSc (106).	\
		IGFBP-5	TGF-β	IGFBP-5 promotes EMT in SSc through TGF-β signalling (105).	\
		\	TGF-β/Akt–NF-κB–IKK	Withaferin A downregulates the Akt–NF-κB–IKK inflammatory signalling axis as well as TGF-β- and Smadmediated EMT (112).	Withaferin A
		\	NOTCH	The NOTCH signalling pathway participates in EMT in SSc (108).	\
		\	Wnt/β-catenin	The Wnt/β-catenin signalling pathway participates in EMT in SSc (107).	\
		SFRP4	\	SFRP4 may serve as a potential EMT-associated molecular marker during immune-driven fibrotic processes in SSc (109).	\
		\	\	The findings support the involvement of EMT in the progression of SSc (102).	\
	Morphea	\	\	In morphea, eccrine sweat gland epithelial cells undergo EMT (114).	\
Other Chronic Inflammatory Diseases	Hidradenitis suppurativa	\	\	The SYK inhibitor fostamatinib suppresses EMT in hidradenitis suppurativa (121).	fostamatinib
	Leprosy	\	TGF-β	In multibacillary leprosy, TGF-β-mediated EMT is implicated (123).	\
	Lymphedema	\	TGF-β	In lymphedema, sustained TGF-β1 signalling in the tissue microenvironment promotes EMT through the TGF-βR2 and Smad axes (135).	\
		\	TGF-β	Pirfenidone and an IL-13 neutralizing antibody blocked TGF-β and Smad mediated EMT, and showed efficacy in a clinical cohort of eight patients (12).	Pirfenidone/IL-13 neutralizing antibody
	RISF	Radiation	TGF-β	Radiation activates TGF-β1-associated signalling and promotes EMT (118).	\
		Radiation	Wnt/β-catenin	Radiation activates the Wnt/β-catenin pathway in skin cells as well as contributes to EMT (119).	sLRP6E1E2
	Rosacea	Pathway	TGF-β	An IL-17A neutralizing antibody suppresses the CXCL5/CXCR2 axis and downregulates TGF-β1- and Smad-mediated EMT in rosacea (127).	IL-17A neutralizing antibody

#### TGF-β signaling pathway

The TGF-β signaling pathway represents one of the best-characterized and most extensively validated signaling axes implicated in EMT. Ligand binding to cognate TGF-β receptors triggers a highly coordinated intracellular signaling network comprising canonical Smad-dependent and non-canonical Smad-independent pathways. The magnitude, duration, and specificity of these signals are tightly controlled by diverse regulatory mechanisms (45). In canonical TGF-β signaling, activated TGF-βRI phosphorylates the receptor-regulated Smads, Smad2 and Smad3. These phosphorylated Smads subsequently associate with Smad4 and translocate into the nucleus, where they orchestrate the transcriptional activation of key EMT-TFs, including SNAI1, SNAI2, TWIST1, and ZEB1. Beyond canonical Smad signaling, TGF-β1 also activates diverse non-canonical pathways, such as PI3K/AKT/mTOR, MEK/ERK, and ERK1/2 signaling, thereby shaping multiple cellular responses, TGF-β signaling also extends beyond the conventional Smad and non-Smad axes, as it can interact with multiple molecular complexes to regulate EMT-related pathways, including Notch, Wnt signaling (46).

#### WNT signaling pathway

The Wnt/β-catenin pathway is an evolutionarily conserved signaling axis that contributes importantly to EMT. Activation by Wnt ligands, including Wnt3a and Wnt1, promotes β-catenin accumulation and nuclear translocation, where β-catenin partners with TCF/LEF transcription factors to drive EMT- and profibrotic gene expression (47). In epithelial cells, E-cadherin normally limits this process by retaining β-catenin at adherens junctions; however, E-cadherin downregulation during EMT relieves this restraint and amplifies β-catenin-mediated transcriptional signaling (48).

#### NOTCH signaling pathway

Notch signaling represents an evolutionarily conserved cell–cell communication pathway that governs interactions between neighboring cells and has been implicated in fibrotic remodeling (49). Following ligand engagement, Notch receptors undergo sequential proteolytic cleavage by ADAM-family metalloproteinases, namely a disintegrin and metalloproteinase (ADAM), particularly ADAM17, and γ-secretase. This process releases the Notch intracellular domain (NICD), which subsequently translocates into the nucleus and associates with CSL (C-repeat/DRE binding factor 1 [CBF1]/suppressor of hairless/Lag1), thereby regulating the expression of EMT-TFs (50–52). Apart from the NICD-mediated direct transcriptional effects, Notch signaling also shapes EMT indirectly through functional crosstalk with TGF-β, NF-κB, and β-catenin signaling axes (49).

#### HIF - 1 signaling pathway

Hypoxia-inducible factor-1 (HIF-1) is a pivotal regulator of hypoxia-driven transcriptional programs and is increasingly recognized as an important contributor to fibrotic remodeling and tumor biology. Under normoxic conditions, HIF-1α is rapidly hydroxylated at defined proline residues by oxygen-dependent prolyl hydroxylase domain enzymes (PHDs), which targets it for ubiquitin–proteasome-mediated degradation. When oxygen becomes limited, PHD activity is suppressed, allowing HIF-1α to evade degradation and accumulate. Stabilized HIF-1α then translocates to the nucleus, where it heterodimerizes with HIF-1β. The resulting HIF-1 complex binds to hypoxia-responsive elements (HREs) and promotes the transcription of hypoxia-responsive genes (53). HIF-1α facilitates EMT by inducing EMT-TFs and activating EMT-related signaling cascades. Notably, HIF-1α functionally interacts with the TGF-β/Smad pathway, with both signaling axes converging on key EMT regulators such as Snail1, ZEB1, TWIST1, and PKM2, ultimately promoting the acquisition of a mesenchymal phenotype (54). Under hypoxic conditions, Notch facilitates the recruitment of HIF-1α to the lysyl oxidase (LOX) promoter, thereby enhancing hypoxia-induced LOX expression and promoting Snail-1 expression (55). In cutaneous fibrosis, vascular dysfunction, capillary rarefaction, impaired perfusion, and excessive ECM deposition may reduce local oxygen availability, creating a hypoxic fibrotic microenvironment (56). Evidence from systemic sclerosis and pathological scars also suggests hypoxia-related changes and increased HIF-1α activity in fibrotic skin (57, 58). Thus, hypoxia/HIF-1α signaling may represent an important microenvironmental regulator of epithelial plasticity and fibrotic remodeling in cutaneous fibrosis.

### EMT in cutaneous fibrotic diseases

**Figure 2:**
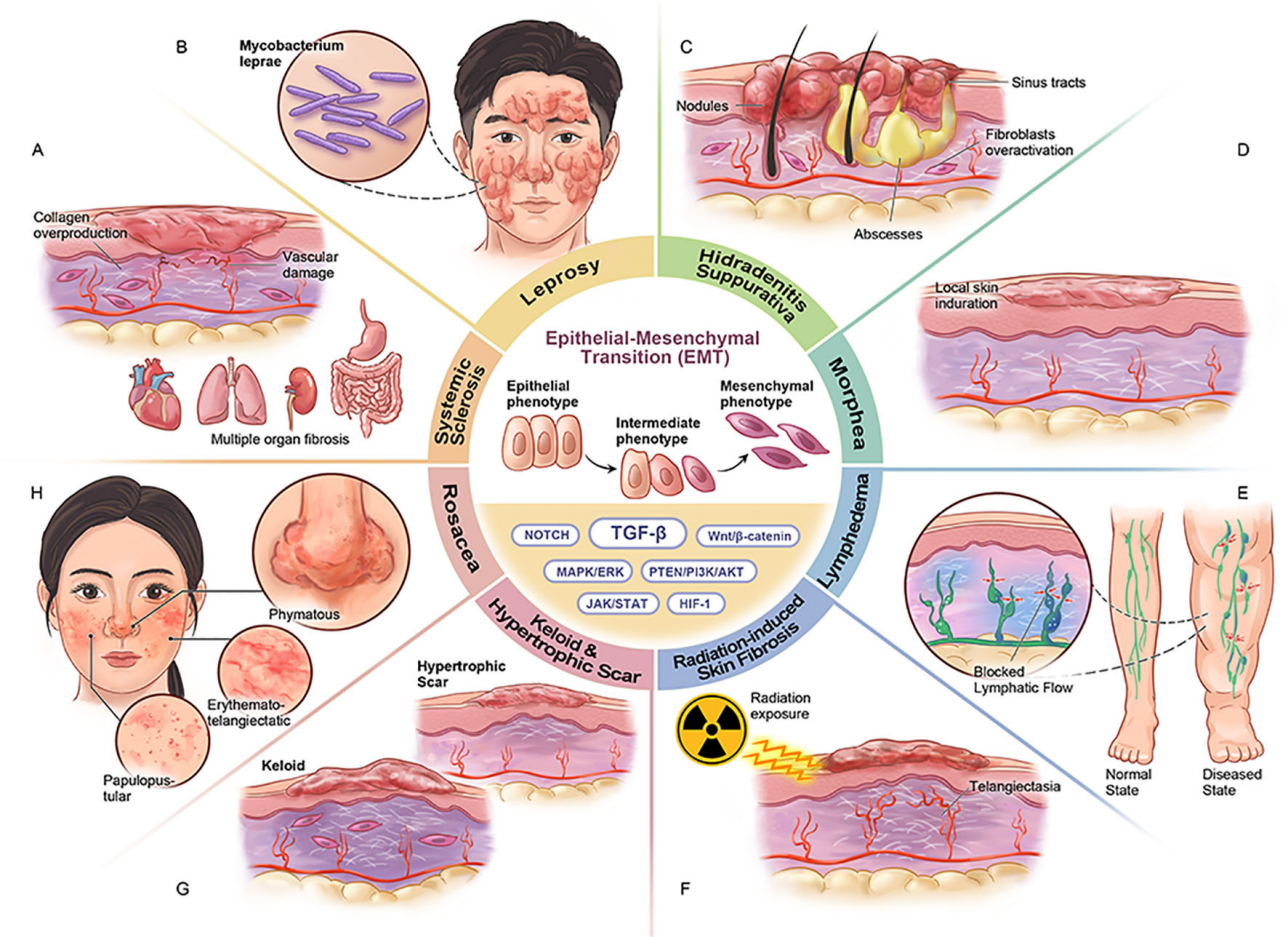
Regulation of EMT in cutaneous fibrotic diseases. Multiple signalling pathways regulate EMT across diverse diseases. Among them, the canonical TGF-β pathway serves as a key regulator, and other key regulatory pathways include HIF-1, PTEN/PI3K/AKT, Wnt/β-catenin, MAPK/ERK, NOTCH, and JAK/STAT signalling. **(A)** Systemic sclerosis features fibrotic involvement of the skin and internal organs; **(B)** leprosy is caused by *Mycobacterium leprae*, with secondary skin fibrosis in late stages; **(C)** hidradenitis suppurativa can result in dermal fibrosis in advanced stages; **(D)** morphea presents with localized fibrosis of the skin and subcutaneous tissue; **(E)** lymphedema leads to fibrotic skin changes in chronic stages; **(F)** radiation-induced skin fibrosis occurs after radiotherapy; **(G)** pathological scars include hypertrophic scars confined to the original wound and keloids that extend beyond and progress; **(H)** rosacea may lead to skin fibrosis due to chronic inflammation.

Cutaneous blood and lymphatic vascular networks are not only essential for tissue repair, immune surveillance, and fluid homeostasis, but also represent important microenvironmental regulators of skin fibrosis. In cutaneous fibrosis, blood capillaries may exhibit endothelial dysfunction, increased permeability, capillary rarefaction, aberrant angiogenesis, and impaired perfusion, collectively promoting local hypoxia and inflammatory cell infiltration (59). In parallel, lymphatic capillary dysfunction can compromise the clearance of interstitial fluid, antigens, and inflammatory mediators, leading to tissue edema, persistent inflammation, and disturbed immune cell trafficking (60, 61). Although direct evidence linking vascular or lymphatic capillary abnormalities to EMT induction in cutaneous fibrosis remains limited, these changes may indirectly support EMT-like epithelial plasticity by sustaining hypoxia, inflammation, and profibrotic cytokine signaling. The major EMT-related mechanisms and signalling pathways involved in different cutaneous fibrotic diseases are summarized in **Figure 2**.

In cutaneous fibrosis, the relationship between EMT and excessive ECM deposition should not be viewed solely as a process in which epithelial cells acquire fibroblast- or myofibroblast-like phenotypes. Rather, it also involves changes in epithelial secretory function during partial EMT (pEMT). Emerging evidence suggests that pEMT cells may preserve selected epithelial traits while adopting a profibrotic secretory profile. This altered secretome can promote the release of profibrotic mediators and reinforce epithelial-fibroblast communication, thereby contributing to ECM accumulation. Importantly, activated dermal fibroblasts and myofibroblasts remain the dominant producers of ECM in fibrotic skin; EMT-related signals may instead shape the profibrotic milieu by enhancing fibroblast activation and matrix remodeling (62, 63). Activated fibroblasts are marked by heightened bioenergetic and biosynthetic requirements, accompanied by metabolic rewiring that includes increased glycolytic flux, enhanced glutamine utilization, activation of serine-glycine and proline metabolic pathways, and changes in mitochondrial function and redox balance (64). Together, these adaptations supply the energy and building blocks needed for collagen synthesis, maturation, and secretion, ultimately supporting persistent ECM deposition. Excessive ECM deposition reshapes the tissue mechanical microenvironment by altering matrix stiffness, compressive forces, and shear stress, thereby modulating EMT plasticity through mechanotransduction. Epithelial cells sense these mechanical cues primarily through integrin–focal adhesion complexes and the E-cadherin–catenin adherens junction complex, with the cytoskeleton acting as a key intracellular conduit. These inputs are then translated into the activation of mechanotransductive pathways, including FAK/Src, PI3K/AKT, RhoA/ROCK, YAP/TAZ, and MRTF-A signaling. Activation of these pathways promotes the expression of EMT-associated transcription factors such as TWIST1, SNAI1, and ZEB1, resulting in loss of epithelial polarity, disruption of intercellular junctions, and increased migratory capacity (65, 66).

### Pathological scars

Scarring is named as the morphological and histopathological changes happening inside normal skin, which are produced as a reaction against various types of injuries. It is considered to be an indispensable component belonging to the wound-healing process. During tissue repair, disrupted regulation of ECM turnover, encompassing synthesis and breakdown, which is driven by multiple cellular and molecular factors, can lead to ECM deposition and the development of pathological scar tissue (67). Scars are generally categorized into three types: physiological scars, hypertrophic scars (HSs), and keloids (68). hysiological scarring is a normal, well-organized, and self-limiting result coming from tissue repair. In contrast, both HSs and keloids are recognized as pathological forms of scarring, yet they show different clinical and biological characteristics. HSs are limited within the borders of the original wound and often have partial spontaneous shrinkage. Contrarily, keloids spread outside the initial injury region and keep growing continuously. Hence, keloids are commonly regarded as a fibrotic skin condition that has tumor-like properties.

#### Keloids

Hahn et al. (69) analysed gene expression profiles of keratinocytes derived from keloid tissue and reported reduced expression of desmosomal proteins, along with upregulation of multiple EMT-associated genes. In addition, compared with normal keratinocytes, keloid-derived keratinocytes demonstrated significantly increased invasive capacity *in vitro*. Taken together, these observations implicate EMT as a key contributor to keloid progression.

The induction of EMT inside keloids is controlled by multiple signalling routes, among them, the transforming growth factor-β (TGF-β) route is broadly considered as the canonical adjuster of EMT and has been carried out extensive research.

Yan et al. (70) demonstrated that keratinocytes in keloids undergo EMT through a signalling axis involving TGF-β1 and its downstream effector, phosphorylated Smad3. Activating the TGF-β1/Smad3 pathway results in the downregulation of epithelial markers and the acquisition of mesenchymal phenotypic traits. Notably, this axis is tightly regulated by multiple upstream modulators and downstream effectors. Homeodomain-interacting protein kinase 2 (HIPK2), a serine/threonine kinase, has been identified as an upstream regulator of fibrotic signalling. Zhao et al. (71) reported that HIPK2 is upregulated in keloids and promotes EMT in keratinocytes by activating the TGF-β1/Smad3 pathway, as evidenced by reduced E-cadherin and ZO-1 levels and elevated vimentin and fibronectin expression. Similarly, phosphodiesterase 4 (PDE4), another upstream regulator, contributes to EMT by degrading cyclic adenosine monophosphate (cAMP) (72), thereby exerting proinflammatory effects and promoting fibroblast proliferation and myofibroblast differentiation. In keloids, PDE4B is overexpressed, and stimulation with TGF-β1 induces the upregulation of both PDE4B and phosphorylated Smad3, thereby facilitating EMT progression (73).

Beyond the canonical TGF-β/Smad pathway, several noncanonical signalling pathways have been implicated in promoting EMT in keloids. Ma et al. (58) demonstrated that hypoxic conditions increase the expression of HIF-1α, which enhances the invasive capacity of keloid-derived keratinocytes and promotes EMT. The authors further proposed that this hypoxia-driven EMT program may underlie the ability of keloids to extend beyond the original wound margins. Pyruvate kinase M2 (PKM2), in addition to its metabolic role, functions as a transcriptional coregulator under hypoxia, thereby amplifying HIF-1α–dependent signalling. Lei et al. (74) revealed that hypoxia induces EMT in keloids via the HIF-1α/PKM2 signalling axis, as indicated by the downregulation of E-cadherin expression, upregulation of vimentin expression, and increased cell motility. Multiple additional pathways have also been shown to contribute to EMT regulation in keloids. Wnt family member 5A (WNT5A), a component of the noncanonical Wnt signalling pathway, promotes interleukin-6 (IL-6) secretion from activated keloid fibroblasts. In turn, IL-6 stimulates the JAK/STAT signaling cascade in keratinocytes, thereby inducing EMT-associated gene expression (75). Vitamin D signalling through its receptor, the vitamin D receptor (VDR), has antifibrotic and anti-inflammatory effects. Moreover, the vitamin D pathway has been implicated in the negative regulation of EMT. The expression of cytochrome P450 family 24 subfamily A member 1 (CYP24A1), a key enzyme responsible for vitamin D inactivation, is markedly upregulated at the protein level in keloids, leading to excessive attenuation of vitamin D signalling and thereby promoting EMT progression (76). The inhibition of CYP24A1 using VID400 or supplementation with vitamin D has also been indicated to reduce the levels of profibrotic and EMT-related genes in keloid tissue (76). Zhang et al. (77) further reported that the transcription factor encoded by ETS-related gene 1 (ERG) directly binds to and activates secreted frizzled-related protein 1 (SFRP1), thereby inhibiting Wnt3a/β-catenin signalling, promoting apoptosis in keloid fibroblasts, and reducing EMT and fibrotic responses. Recent studies have also identified the PTEN/PI3K/AKT signalling axis as a regulator of EMT-associated gene expression in keloid-derived keratinocytes (78). Cellular stress can activate extracellular signal–regulated kinase (ERK), a key effector in the mitogen-activated protein kinase (MAPK) cascade (79). ERK phosphorylation has been found to increase HIF-1α expression and amplify the EMT transcriptional program. Kim et al. (80) reported that ERK activation promotes cytoskeletal remodelling, as reflected by increased levels of EMT markers such as vimentin and α-SMA. Furthermore, ERK signalling induces expression of MMP-2 and MMP-9, further facilitating ECM remodelling and enhancing cell migration.

Substantial progress has been made in targeted therapeutic strategies directed at the EMT process in keloids. A growing number of inhibitors have been demonstrated to attenuate fibrosis progression through the selective blockade of key EMT-related signalling pathways.

Pirfenidone, a small-molecule agent approved as a therapeutic option for pulmonary fibrosis, has been reported to suppress TGF-β1–mediated EMT-associated gene expression in primary keloid-derived keratinocytes while concurrently inhibiting cell proliferation and migration (81). Similarly, in primary human keratinocytes, Kelulut honey, a natural product from Malaysia, alleviates cutaneous fibrosis by blocking TGF-β1–induced EMT (82). Consistent with these findings, Hahn et al. found that the SMAD2/SMAD3 antagonist SB525334 effectively inhibits EMT progression in primary keloid-derived keratinocytes (83). In addition, in primary keratinocytes isolated from the epidermis of keloid tissues, the PDE4B inhibitor roflumilast suppresses TGF-β1–induced Smad3 phosphorylation and attenuates mesenchymal differentiation (73). Hyperbaric oxygen therapy (HBOT), which is widely used to promote wound healing, reduce inflammation, and enhance tissue survival (84), has also been evaluated for keloid treatment. Zhang et al. (85) enrolled 18 patients with keloids and randomized them into HBOT-treated and untreated groups. Compared with controls, HBOT improved the hypoxic microenvironment within keloid tissue, reduced vimentin and HIF-1α levels while elevating E-cadherin and ZO-1 expression, indicating that compared with controls, HBOT can effectively attenuate EMT in keloids. In addition, metformin suppresses hypoxia-induced EMT by targeting the HIF-1α/PKM2 signalling axis. Moreover, β-sitosterol modulates the activity of the PTEN/PI3K/AKT signalling pathway to suppress cell viability, invasion, and migration. These effects are accompanied by reduced expression of vimentin and snail and increased expression of ZO-1 and E-cadherin (78). ERK pathway inhibition represents another effective approach. The selective ERK inhibitor SCH772984 blocks hypoxia-induced EMT, while U0126 and roflumilast inhibit ERK1/2 phosphorylation to achieve similar effects (73, 83). Finally, Qiu et al. (86) identified ribavirin as a potent small-molecule compound capable of reversing EMT in keloid cells through database-driven screening and confirmed its efficacy *in vitro*, highlighting its promising therapeutic potential.

Overall, EMT in keloids should be viewed not as the consequence of a single signaling pathway, but as the product of multiple profibrotic signals acting in concert, particularly TGF-β, hypoxia, and inflammation-related pathways. Despite differences in the upstream molecular events emphasized across studies, these signals are likely to converge on a shared EMT transcriptional program centered on the SNAIL, ZEB, and TWIST families, ultimately promoting epithelial plasticity, ECM remodeling, and persistent fibrotic progression in keloids.

#### Hypertrophic scarring

In contrast to keloids, HSs remain confined to the original wound edges and often exhibit partial spontaneous regression over time. Yan et al. (87) were the first to report significant epidermal thickening in HSs, accompanied by FSP1 (fibroblast-specific protein 1) expression, a marker present in both newly formed and differentiated fibroblasts and widely used in previous studies to confirm EMT (88), and vimentin in epidermal cells adjacent to the basement membrane zone. Concurrent upregulation of the expression of the EMT-TFs Snail1 and Twist1 further supports the presence of an EMT program. Impaired epidermal barrier function may itself act as a potential upstream driver of pathological epidermal phenotypes in hypertrophic scar skin (89). Following injury, disruption of the epidermal barrier increases transepidermal water loss, exposing keratinocytes to a persistently low-hydration microenvironment accompanied by increased pericellular solute concentration and local osmotic stress. These changes may activate stress-responsive pathways such as p38 and JNK, thereby amplifying abnormal epidermal responses (90, 91). Meanwhile, local ionic homeostasis may also be disturbed, particularly through increased Na^+^ flux mediated by epithelial sodium channels (ENaC). The significance of this alteration lies not simply in sodium entry itself, but in its ability to enhance inflammatory and paracrine activity in keratinocytes, promoting the release of mediators such as cyclooxygenase-2 and prostaglandin E2. This, in turn, may strengthen keratinocyte–fibroblast crosstalk, promote fibroblast activation, and contribute to a profibrotic microenvironment that supports pathological scar formation (92). Consistent with this barrier-centered mechanism, hydration status also represents a critical determinant of wound healing. Epithelial barrier disruption following injury results in a reduced hydration environment. Keratinocytes exposed to low-hydration conditions exhibit increased Snail expression and decreased E-cadherin levels, leading to increased β-catenin–TCF/LEF-1 transcriptional activity and the promotion of EMT in HSs (93). Slit, a secreted guidance protein involved in cell migration and adhesion, has also been implicated in this process. Slit induces the phosphorylation of both SMAD-dependent components (SMAD2, SMAD3, and SMAD1/5/8) and SMAD-independent proteins (TAK1, JNK, ERK1/2, and p38), thereby activating multiple signalling cascades that enhance EMT and promote ECM accumulation in HSs (94). Exosomes, membrane-bound extracellular vesicles that mediate intercellular communication by transporting proteins and genetic material (95), have been increasingly recognized as contributors to fibrosis pathogenesis. Cui et al. (96) suggested that exosomes derived from HSs can induce EMT in recipient cells by downregulating E-cadherin expression and upregulating N-cadherin and vimentin expression via the activation of TGF-β downstream of the SMAD and TAK1 signalling pathways, highlighting their profibrotic potential. Additionally, the expression of S100A8 and S100A9, members of the S100 calcium-binding protein family involved in chronic inflammation, is associated with increased EMT activity. PU.1, a PU box-binding transcription factor, has been shown to induce S100A8/A9 expression, facilitating Snail phosphorylation and nuclear translocation and thereby enhancing EMT and the invasive capacity of dermal keratinocytes in post-burn scarring (97).

Several therapeutic strategies have been shown to effectively target EMT in HSs. Extracorporeal shock wave therapy (ESWT), a noninvasive physical modality, has recently been applied in HSs management. Cui et al. (98) reported that, in primary dermal fibroblasts derived from human hypertrophic scar tissue, ESWT significantly lowered EMT-associated marker levels, including TGF-β1, α-SMA, and Twist1—while elevating expression of the epithelial marker E-cadherin in HS tissue. These findings suggest that EMT inhibition may partly underlie the antiscarring effects of ESWT. Additionally, botulinum toxin type A (BTXA) has demonstrated efficacy in treating HSs. In murine L929 fibroblasts, BTXA suppresses TGF-β1 signalling, thereby reducing fibroblast viability, inducing apoptosis, and downregulating the expression of EMT-associated molecules. It also inhibits fibroblast phenotypic transition by attenuating phosphatase and tensin homologue (PTEN) methylation and PI3K/Akt pathway activation (99). Furthermore, the antifungal agent hydroxypyridone has emerged as a novel candidate for HS treatment. This compound targets myofibroblasts, reducing ECM production and inhibiting EMT in keratinocytes, thereby exerting broad antifibrotic effects (100).

Although therapeutic strategies targeting key EMT regulators and signalling pathways have shown promise in suppressing aberrant fibroblast activation and excess ECM accumulation in keloids and HSs, both *in vitro* and in animal models, their clinical translation remains limited. Most studies to date are still in the preclinical stage, and there is a notable lack of clinical trials that are rigorously designed and adequately powered randomized controlled trials to rigorously assess clinical efficacy and long-term safety in patients. In addition, practical challenges such as drug delivery routes, target specificity, delivery efficiency, manufacturing feasibility, and cost continue to impede the broad clinical implementation of EMT-targeted therapies.

Taken together, EMT in hypertrophic scars does not appear to be driven by a single pathway, but is more likely shaped by the combined effects of persistent post-injury inflammation, exosome-mediated intercellular dysregulation, and reduced local hydration. Although the upstream mechanisms highlighted across studies differ, signals including Slit1, TGF-β/SMAD, PU.1/S100A8/A9, and β-catenin–TCF/LEF-1 may converge on a transcriptional regulatory network centered on the Snail, ZEB, and Twist families, thereby promoting epithelial plasticity and pathological scar formation.

### Autoimmune diseases

Cutaneous fibrosis associated with autoimmune diseases is driven by chronic immune activation following the breakdown of immune tolerance. Imbalance in innate and adaptive immune responses leads to persistent production of pro-fibrotic cytokines, which promote aberrant fibroblast activation and the accumulation of myofibroblasts. Growing evidence suggests that the chronic inflammatory milieu characteristic of autoimmune diseases serves as a powerful EMT trigger and consequently worsens pathological fibrosis (101).

#### Systemic sclerosis (SSc)

SSc is a rare, clinically heterogeneous autoimmune connective tissue disorder featuring fibroproliferative vasculopathy and immune dysregulation, accompanied by progressive skin and visceral fibrosis that may ultimately lead to multiorgan dysfunction and failure. Nakamura et al. (102) performed immunohistochemical analysis of forearm skin biopsy specimens from three patients with diffuse cutaneous systemic sclerosis and detected the expression of the EMT-TFs SNAI1 and TWIST1 in eccrine gland structures. These findings suggest possible EMT-marker expression in eccrine gland epithelial structures in SSc skin.

Research on the emergence and progression of fibrosis in SSc has predominantly focused on TGF-β signalling, which serves as a central regulatory axis. Nikitorowicz-Buniak et al. (103) observed that SSc patient skin biopsy samples exhibit elevated levels of phosphorylated SMAD2/3, indicating that cutaneous fibrosis development is driven, at least in part, by TGF-β/SMAD-dependent signalling. Brachyury, an evolutionarily conserved transcription factor and a robust inducer of EMT in human cancer cell lines, is markedly expressed in SSc skin, potentially as a result of endogenous TGF-β signalling. Brachyury knockdown lowered COL1A1 levels in dermal fibroblasts derived from healthy donors and patients with SSc; However, it did not significantly affect the expression of major disease-associated EMT markers (104). Insulin-like growth factor-binding proteins (IGFBPs), a family of circulating proteins implicated in fibrosis, are also involved in EMT regulation in SSc. IGFBP-3 and IGFBP-5 show increased levels in SSc patient–derived primary dermal fibroblast cultures and are capable of promoting a fibrotic phenotype *in vitro*. Notably, IGFBP-5 promotes EMT through TGF-β pathway activation, thereby enhancing fibroblast activation and fibrosis progression (105). Additionally, Bakhshi et al. (106) reported that TGF-β stimulation markedly increases the RAF and RalGDS expression in SSc, suggesting that aberrant amplification of the TGF-β–Ras–RAF–ERK and RalGDS pathways enhances EMT-associated transcriptional programs and further contributes to fibrosis.

Apart from TGF-β signaling, several other EMT-associated signalling cascades, such as the NOTCH and WNT pathways, have been linked to the onset and progression of SSc (107, 108). Tinazzi et al. (109) reported that secreted frizzled-related protein 4 (SFRP4) is significantly upregulated in SSc and is localized to basal epidermal epithelial cells, which exhibit a vimentin-positive, caveolin-1–deficient phenotype. *In vitro* studies further demonstrated that SFRP4 levels rise alongside EMT markers such as COL1A1, Snail, and vimentin, supporting its involvement in EMT activation. Moreover, SFRP4 expression is strongly correlated with that of other WNT-related genes, including WIF1 and WNT2, suggesting that SFRP4 may serve as an indicator of aberrant WNT signalling activity in SSc.

Recent studies have identified LG283, a curcumin-derived small molecule, that suppresses EMT by inhibiting the TGF-β/Smad/Snail axis and may represent a promising therapeutic candidate for SSc (110). In addition, Diddiet al. (111) showed that nimbolide, a triterpenoid derived from the Indian medicinal species *Azadirachta indica*, displays antifibrotic and anti-inflammatory effects in a preclinical bleomycin-induced murine model of scleroderma. Nimbolide significantly disrupted TGF-β/Smad signalling, thereby blocking EMT and attenuating ECM deposition. Forkhead box O3a (FoxO3a), a member of the FoxO transcription factor subfamily, has also been been linked to fibrosis regulation. Bale et al. (112) showed that withaferin A (WFA), a highly oxygenated steroidal lactone and major bioactive constituent of *Withania somnifera*, effectively attenuates dermal fibrosis in a 28-day bleomycin-induced murine model of experimental scleroderma by restoring FoxO3a activity and suppressing the Akt–NF-κB–IKK inflammatory signalling cascade. These effects were associated with the downregulation of TGF-β/Smad-mediated EMT programs and a reduction in the fibrotic burden. Despite the growing number of compounds capable of inhibiting EMT in experimental SSc models, most studies remain limited to preclinical or exploratory phases. Currently, no EMT-targeted therapies have been conclusively validated as stable, efficacious, and clinically applicable for the routine treatment of SSc.

Sclerodermatous chronic graft-versus-host disease (sclGVHD) is a fibrotic subtype of chronic GVHD characterized predominantly by cutaneous sclerosis and dermal fibrosis. Although its etiology differs from that of SSc, with sclGVHD arising from allogeneic immune reactions following hematopoietic stem cell transplantation, whereas SSc represents an autoimmune fibrotic disorder, the two conditions share several pathological features of skin fibrosis, particularly excessive ECM deposition and dermal sclerosis. Accordingly, sclGVHD is often regarded as an SSc-like, immune-driven model of cutaneous fibrosis and may therefore provide supportive insights into mechanisms relevant to SSc-associated fibrotic remodeling (113). At present, however, direct evidence linking EMT to cutaneous GVHD remains limited. Tinazzi et al. reported increased ECM deposition and elevated SFRP4 expression in the skin of patients with sclerodermatous chronic GVHD, with SFRP4-positive cells localized to dermal fibroblasts and the epidermal basal layer. Given that SFRP4 is also upregulated in epithelial cells undergoing TGF-β-induced EMT *in vitro*, these findings suggest that EMT-related epithelial plasticity may accompany sclGVHD-associated skin fibrosis. Nevertheless, direct evidence demonstrating bona fide EMT or definitive epithelial lineage conversion in GVHD skin is still lacking (109).

#### Morphea

Morphea is a localized cutaneous fibrotic disorder involving the skin and subcutaneous tissue. Unlike SSc, morphea is characterized by a limited disease extent and lacks systemic vasculopathy or internal organ involvement. Takahashi et al. (114) analyzed skin lesion specimens from six patients with morphea and control skin samples from healthy individuals using histological and immunostaining approaches. They found increased expression of fibrosis-related markers, including TGF-β1, α-SMA, and fibronectin, in the dermis of morphea lesions. Notably, their EMT-focused analysis of eccrine glands showed increased Snail1 expression and reduced E-cadherin expression, together with increased fibronectin expression, suggesting EMT-like changes in eccrine gland epithelial structures. Based on these observations, the authors proposed that epithelial cells within eccrine sweat glands may undergo EMT, migrate into the dermis, and contribute to myofibroblast accumulation, collagen production, and dermal fibrotic remodeling.

Increasing evidence has drawn considerable attention to the potential involvement of EMT in the initiation and progression of skin fibrosis in SSc. Among the various signal pathways, the regulatory network which takes TGF-β as the core is commonly recognized to be a major trigger of EMT and unusual fibroblast activation in systemic sclerosis. Therefore, even though TGF-β-mediated processes have been the key focus in this research field, relatively little research work has been conducted to make clear the functions of other classic EMT pathways, for instance the WNT and NOTCH signal systems, in the development of SSc-related skin fibrosis.

EMT in autoimmune-associated cutaneous fibrosis should not be regarded as the consequence of a single signaling pathway, but rather as a complex pathological program driven by impaired immune tolerance, chronic inflammation, vascular dysfunction, and a TGF-β-rich profibrotic microenvironment. Despite differences in the upstream molecular events emphasized across studies, these signals may ultimately converge on an EMT transcriptional network centered on the SNAIL, ZEB, and TWIST families, thereby enhancing epithelial plasticity, ECM remodeling, and progressive fibrotic change. In addition, autoimmune diseases beyond systemic sclerosis and morphea, particularly systemic lupus erythematosus, may also develop cutaneous fibrosis at later stages; however, the contribution of EMT and its underlying molecular basis in these conditions remain poorly defined. Further investigation of these unresolved questions will be important for broadening our understanding of autoimmune-driven skin fibrosis and for uncovering therapeutic opportunities beyond the classical TGF-β-centered framework.

### Other chronic inflammatory diseases

In parallel with cutaneous fibrosis associated with scarring and autoimmune diseases, a range of chronic inflammatory skin disorders, such as rosacea, hidradenitis suppurativa, leprosy, chronic lymphedema, and radiation-induced skin fibrosis (RISF), can also develop fibrotic changes during advanced stages of disease. Accumulating evidence suggests that EMT represents a common and central mechanism driving fibrosis progression across these diverse inflammatory conditions.

#### RISF

Radiotherapy remains a cornerstone of cancer treatment; however, roughly 30% to 70% of patients experience persistent cutaneous injury that may progressively evolve into RISF, resulting in functional impairment, chronic pain, and decreased quality of life (115–117). Dodson et al. (118) reported that the activation of a TGF-β1-associated profibrotic program in irradiated skin is accompanied by the upregulation of levels of EMT markers including SNAI1, SNAI2, and TWIST1, consistent with EMT activation. Lee et al. (119) reported that radiation stimulates the Wnt/β-catenin pathway in keratinocytes, which in turn promotes TGF-β/Smad signalling and enhances the production of collagen, including COL1A1 and COL3A1. In parallel, radiation-induced downregulation of E-cadherin, together with upregulation of vimentin and TWIST, collectively drives EMT. Notably, sLRP6E1E2, a Wnt ligand antagonist, inhibited β-catenin nuclear translocation, thereby reducing EMT and collagen deposition and alleviating cutaneous fibrosis.

#### Hidradenitis suppurativa

Hidradenitis suppurativa is a chronic inflammatory skin disease marked by recurrent inflammatory nodules and abscesses that, in later stages, progress to the formation of epithelialized tunnels and scarring (120). Flora et al. (121) reported that hidradenitis suppurativa lesions exhibit prominent transcriptional signatures of inflammation and ECM remodelling, accompanied by the reprogramming of dermal fibroblast subpopulations. Notably, the expression of EMT markers, including TWIST1, ZEB1, Snail, Slug, and N-cadherin, is elevated in lesional tissue, particularly within tunnel-associated regions, suggesting that EMT contributes to tunnel formation, maintenance, and localized fibrotic remodelling. Spleen tyrosine kinase (SYK), a nonreceptor tyrosine kinase, has been implicated in profibrotic processes across various fibrotic diseases (122). Treatment with the SYK inhibitor fostamatinib in hidradenitis suppurativa was shown to downregulate the expression of fibrosis- and EMT-associated genes, further supporting the roles of inflammation-driven fibroblast remodelling and EMT as critical and potentially targetable mechanisms in the pathological progression of this condition.

#### Leprosy

Leprosy, caused by *Mycobacterium leprae*, is a long-term infectious condition characterized by persistent inflammation and immune dysregulation, which may result in sensory loss, deformities, and disability. At advanced stages, secondary fibrosis and scar formation may also occur. Clinically, leprosy can be classified into multibacillary (MB) and paucibacillary (PB) forms. Leal-Calvo et al. (123) analyzed bulk RNA-seq profiles of human leprosy skin lesion biopsies and reported that the expression of genes related to TGF-β-driven EMT was significantly increased in MB lesions. In these whole-lesion transcriptomes, genes associated with keratinocyte differentiation, cornification, and epidermal development were inversely correlated with EMT- and mesenchymal-related gene expression. Moreover, EMT signalling activity increased stepwise from healthy skin to PB and then to MB lesions. These findings suggest that EMT may contribute to impaired keratinization in MB leprosy, thereby weakening the epidermal barrier and potentially promoting bacterial persistence and dissemination. However, the authors emphasized that the causal relationship underlying these observations remains to be validated in functional studies.

#### Rosacea

A chronic inflammatory skin disease, rosacea has multifactorial origins, driven by genetic susceptibility and environmental triggers, innate immune imbalance, neurovascular changes, and host–microbiota interactions in the skin. Changes in fibrotic skin may develop during disease progression (124, 125). Experimental studies have shown that prolonged intradermal administration of the bioactive cathelicidin-derived peptide LL37 in mice induces rosacea-like lesions accompanied by fibrotic features (126). Building on these observations, Zhang et al. (127) reported that in an LL37-induced rosacea-like experimental model, chronic LL37 stimulation activated EMT via the TGF-β1/Smad signalling pathway, thereby promoting myofibroblast differentiation and collagen deposition and ultimately contributing to rosacea-associated fibrotic remodeling. Buhl et al. further demonstrated that genes involved in the IL-17 signalling pathway, including IL17A, CXCL5, and its receptor CXCR2, are significantly upregulated in rosacea (128). Using an IL17A-neutralizing antibody, the authors showed that blockade of IL17A attenuates inflammation and angiogenesis by suppressing CXCL5 and CXCR2 activation while simultaneously inhibiting EMT and fibrotic progression through downregulation of TGF-β1 signalling.

#### Lymphedema

Lymphedema is a chronic, progressive disorder resulting from impaired lymphatic drainage or defective lymphangiogenesis and is commonly associated with tissue swelling, persistent inflammation, adipose deposition, and fibrosis, ultimately leading to a substantial decline in quality of life (129–134). Will et al. (135) demonstrated that within the chronic inflammatory microenvironment of lymphedema, sustained TGF-β1 signalling induces EMT via the TGFBR2-to-Smad pathway, as evidenced by reduced ZO-1 and E-cadherin levels, along with increased N-cadherin and α-SMA levels, accompanied by markedly augmented cell motility and invasiveness. Subsequently, Park et al. (12) confirmed that basal epidermal keratinocytes display EMT-like features and invade the dermis in lymphedema. In this process, the TGF-β-to-Smad signalling cascade serves as a central regulator. Pharmacological intervention with pirfenidone was shown to block EMT and restore epithelial characteristics in EMT-like cells. Moreover, accumulating evidence suggests that the suppression of T helper type 2 (Th2) cytokines reduces TGF-β production in secondary lymphedema and other fibrotic disorders (136). In a clinical cohort, the expression of IL-13, a key Th2 cytokine, was shown to induce keratinocyte EMT through the upregulation of TGF-β signalling. In a pilot study involving eight patients with secondary lymphedema who received anti-Th2 immunotherapy, treatment with an IL-13-neutralizing antibody resulted in a marked reduction in the number of vimentin-positive and phosphorylated Smad3-positive keratinocytes in the epidermis, underscoring the potential value of EMT-focused interventions in lymphedema. Although EMT has emerged as a potential mechanism underlying cutaneous fibrosis, research into its role in other chronic inflammatory diseases remains at a relatively early stage. Current evidence is primarily based on phenotypic observations and correlative analyses. The functional relevance of EMT across distinct phases of disease progression, the identification of its key regulatory factors, and its potential interactions, whether cooperative or antagonistic, with other signalling pathways have yet to be systematically validated through rigorous *in vitro* and *in vivo* studies. These knowledge gaps underscore the need for more comprehensive mechanistic investigations to define the precise role of EMT in chronic inflammatory skin fibrosis.

## Epigenetic regulation of EMT in cutaneous fibrotic diseases

**Figure 3:**
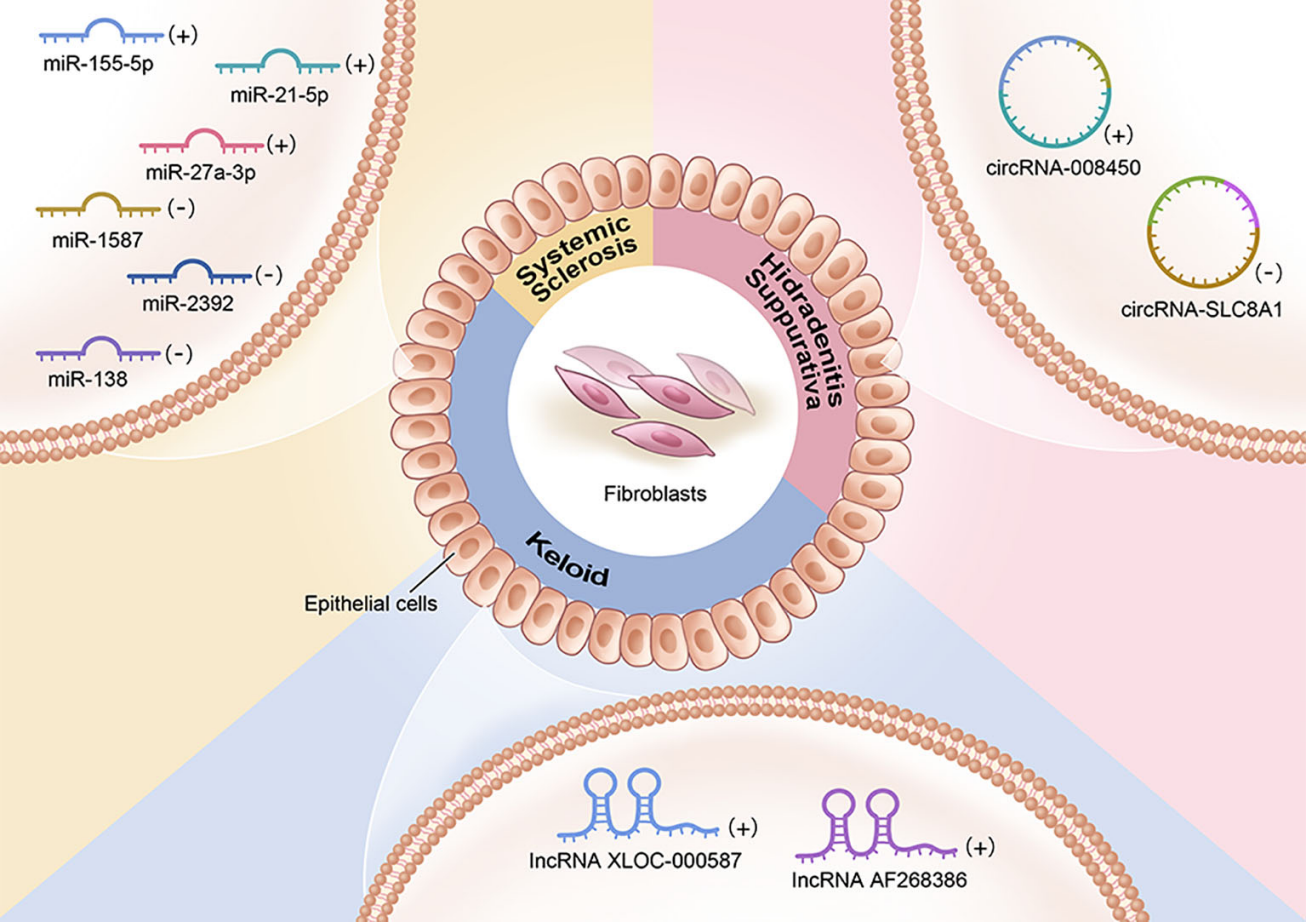
Epigenetic regulation of EMT by ncRNAs in cutaneous fibrotic diseases. In keloids, miR-21-5p, miR-155-5p, miR-1587, and miR-2392 modulate EMT. In systemic sclerosis, miR-138 regulates EMT. In hypertrophic scars, miR-27a-3p regulates EMT, while circ_0008450 and circRNA_SLC8A1 are also involved in EMT modulation. In addition, the lncRNAs XLOC_000587 and AF268386 regulate EMT in keloids. “+” indicates the promotion of EMT, whereas “−” indicates the inhibition of EMT.

Epigenetic modifications encompass diverse regulatory processes, including DNA methylation, histone modification, chromatin remodelling, and epigenetic regulation mediated by noncoding RNAs (ncRNAs). Emerging evidence has demonstrated that epigenetic regulation is crucial for EMT in cancer and fibrotic diseases, highlighting the complexity of this finely tuned regulatory network (137, 138). Accumulating evidence underscores the involvement of ncRNAs, notably microRNAs (miRNAs), circular RNAs (circRNAs), and long noncoding RNAs (lncRNAs), in regulating EMT during cutaneous fibrosis. The ncRNA-mediated regulation of EMT in cutaneous fibrotic diseases is summarized in **Figure 3**. Epigenetic mechanisms governing EMT in cutaneous fibrotic diseases are summarized in **Table 2**.

**Table 2:** Epigenetic mechanisms governing EMT in cutaneous fibrotic diseases.

NcRNA	Disease	Factor	Pathway	Result	Inhibitor
miRNAs	Keloid	miR-21-5p	PTEN/Akt	miR-21-5p directly targets PTEN and alters AKT phosphorylation, thereby further promoting EMT in keloid-derived keratinocytes (141).	\
		miR-155-5p	\	miR-155-5p promotes EMT in keloids (86).	\
		miR-1587 and miR-2392	\	BTXA upregulates miR-1587 and miR-2392 expression, markedly downregulates ZEB2, and thereby suppresses EMT (143).	BTXA
	SSc	miR138	\	miR-138 has been proposed as a potential negative regulator of EMT (144).	\
circRNAs	Hypertrophic scars	circ_0008450	TGF-β	By negatively regulating Runx3, circ_0008450 modulates activation of the TGF-β and Smad signalling pathway, thereby driving EMT (147).	\
		circRNA_SLC8A1	Nrf2–ARE	circRNA_SLC8A1 directly binds miR-27a-3p, activates the Nrf2 and ARE antioxidant response pathway, and suppresses EMT in hypertrophic scars (148).	\
lncRNAs	Keloids	lncRNA XLOC_000587/lncRNA AF268386	\	XLOC_000587 may further regulate EMT by promoting ENAH expression, whereas AF268386 has been proposed to act as an enhancer of DDR2 in the regulation of EMT in keloids (150).	\

### MiRNAs

MiRNAs are endogenous noncoding RNAs of approximately 19–25 nucleotides that modulate gene expression through sequence-specific binding to target messenger RNAs (mRNAs), thereby mediating gene silencing and translational repression (139). Among them, miR-21 is consistently reported to be upregulated in fibrotic skin disorders (140). Yan et al. (141) demonstrated that miR-21-5p is markedly overexpressed in keloid tissues and that modulation of its expression is sufficient to induce or reverse EMT phenotypes in keloid-derived keratinocytes. Notably, changes in miR-21-5p levels were accompanied by alterations in cellular stemness, whereas no significant effects were observed in normal keratinocytes. PTEN and the AKT signalling pathway were identified as direct targets of miR-21 (142). Mechanistically, miR-21-5p suppresses PTEN expression and increases AKT phosphorylation, thereby promoting the acquisition of EMT phenotypes and stemness in keloid keratinocytes. Qiu et al. (86) recognized miR-155-5p as a pivotal miRNA associated with EMT activation and fibrotic progression in keloids through sequencing-based analyses, although direct causal validation remains lacking. Interestingly, Hou et al. (143) reported that the expression of miR-1587 and miR-2392 is significantly downregulated in keloid tissues, while their shared target gene, ZEB2, promotes keloid pathogenesis. BTXA upregulated miR-1587 and miR-2392 expression, resulting in reduced ZEB2 expression and EMT inhibition, and the concomitant induction of apoptosis and autophagy, ultimately attenuating keloid fibrosis. In addition to keloids, miR-138 has been recognized as a negative regulator of EMT signalling. Bayati et al. (144) reported that miR-138 levels are significantly reduced in patients with SSc. On the basis of existing evidence, the authors proposed that miR-138 may exert regulatory effects on EMT by targeting the PI3K/AKT pathway, ZEB2, and extracellular matrix remodelling factors such as MMP2 and MMP9. However, these proposed mechanisms require further validation in larger patient cohorts and through rigorous causal studies.

### CircRNAs

CircRNAs are single-stranded RNAs with covalently closed loops that exert diverse biological functions, including transcriptional regulation, acting as microRNA sponges, and serving as templates for protein translation (145). Runt-related transcription factors (Runxs) are key regulators of developmental processes, and Runx3 has been characterized as a tumour suppressor gene in keloid pathogenesis (146); Chen et al. (147) reported that circ_0008450 is markedly upregulated in the epidermis of keloid tissues, where it exerts its effects by negatively regulating the scar-suppressor gene Runx3. Activation of the circ_0008450–Runx3 axis was shown to modulate TGF-β/Smad signalling, thereby driving EMT, suggesting that circ_0008450 may be a promising candidate for therapeutic modulation in keloids and that circ_0008450 is markedly upregulated in the epidermis of keloid tissues, where it suppresses Runx3, a gene that limits scar formation. Activation of the circ_0008450–Runx3 axis was shown to modulate TGF-β/Smad signalling, thereby driving EMT, suggesting that circ_0008450 may represent a potential therapeutic target for keloid intervention. Jin et al. (148) reported that circRNA_SLC8A1 is significantly downregulated, whereas miR-27a-3p is highly expressed. Functional studies revealed that the overexpression of circRNA_SLC8A1 markedly suppressed EMT. Mechanistically, circRNA_SLC8A1 directly binds to miR-27a-3p and functions as a competing endogenous RNA (ceRNA), thereby alleviating the miR-27a-3p–mediated repression of nuclear factor erythroid 2–related factor 2 (Nrf2), a member of the cap’n’collar family of transcription factors that activates antioxidant response elements (AREs). Activation of the Nrf2–ARE antioxidant pathway subsequently inhibits EMT and excessive collagen deposition in hypertrophic scars, ultimately attenuating fibrosis progression.

### LncRNAs

lncRNAs are noncoding RNA molecules longer than 200 nucleotides that regulate gene expression through diverse mechanisms and can either activate or suppress EMT programs (149). ENAH is an actin-regulatory protein involved in cell motility and adhesion and has been implicated in EMT during tumour progression, where it may contribute to enhanced proliferation and distant metastasis. The lncRNA XLOC_000587 is located upstream of ENAH; on the basis of sequencing analyses, Chen et al. (150) proposed that XLOC_000587 may promote EMT-associated signalling pathways by upregulating ENAH expression, thereby facilitating EMT and the progression of keloid invasion. Moreover, discoidin domain receptor 2 (DDR2) functions as a collagen-activated receptor tyrosine kinase and a key regulator of EMT. Previous studies have predicted that the lncRNA AF268386 functions as an enhancer of DDR2 (151). Accordingly, the authors hypothesized that AF268386 may act as a DDR2-associated enhancer to regulate EMT in keloids and accelerate their invasive behaviour.

Despite accumulating evidence supporting the involvement of ncRNAs, including miRNAs, circRNAs, and lncRNAs, in the regulation of EMT during cutaneous fibrosis, other ncRNA species, such as PIWI-interacting RNAs and small nucleolar RNAs, have thus far been primarily studied in the context of cancer. In these settings, they have been shown to cooperatively construct and modulate complex EMT regulatory networks at multiple levels (152). However, their roles in cutaneous fibrosis remain largely unexplored. Moreover, research on additional epigenetic processes, for example DNA methylation and histone modification, remains scarce in EMT-associated cutaneous fibrosis. The pronounced tissue specificity of epigenetic marks, together with the lack of standardized detection methods and evaluation criteria, further constrains their translational potential as clinical biomarkers or therapeutic targets. Importantly, many current conclusions are derived largely from association analyses based on gene expression, underscoring the need for rigorous functional and mechanistic validation.

## Methodological challenges in detecting EMT in cutaneous fibrosis

Current studies of EMT in fibrosis generally employ multiple complementary approaches, including morphological assessment, transcriptomic and proteomic analyses, immunolocalization, flow cytometry, functional assays, signaling pathway interrogation, single-cell and spatial omics, and lineage tracing. Nevertheless, these methods provide different levels of evidence and all have important limitations. Morphological changes and shifts in EMT-associated marker expression may indicate an EMT-like phenotype, but are insufficient to prove a complete EMT program on their own (22). In tissue specimens, alterations in mRNA or protein expression may also reflect inflammation, changes in cellular composition, or expansion of fibroblast populations, rather than true epithelial lineage conversion. Likewise, although keratinocyte-based *in vitro* models are useful for mechanistic studies of hypoxia- or inflammation-induced EMT, they cannot fully recapitulate the complex microenvironment of human cutaneous fibrosis, including ECM stiffening, mechanical stress, immune infiltration, vascular and lymphatic abnormalities, and epithelial–stromal crosstalk. Thus, *in vitro* evidence should be interpreted as mechanistic support rather than direct proof of EMT *in vivo*. In human tissue sections, co-localization of epithelial and mesenchymal markers by immunofluorescence or immunohistochemistry may provide spatially suggestive evidence, but cannot establish lineage conversion or functional transition, especially because mesenchymal markers such as vimentin, α-SMA, and COL1A1 are not specific to EMT and may arise from adjacent fibroblasts or myofibroblasts (153, 154). Although lineage tracing is one of the most informative approaches for defining cellular origin, it depends on genetic labeling strategies that are largely restricted to experimental models and cannot be readily applied to human tissues (155). Therefore, EMT in cutaneous fibrosis should not be inferred from marker expression alone, but should be supported by functional assays assessing migration, invasion, basement membrane degradation, ECM production, collagen contraction, and epithelial–fibroblast interactions. In the absence of robust evidence for both cellular origin and function, it is premature to conclude from any single method that epithelial cells have fully transitioned into functional myofibroblasts.

## Clinical translation of EMT-targeted therapy in cutaneous fibrosis

At present, clinical studies of EMT-targeted therapies in cutaneous fibrosis remain limited, and the available evidence is summarized. The absence of an approved EMT-targeted therapy likely reflects not only the scarcity of robust clinical data, but also several major translational barriers. One of these is the presence of disease-specific obstacles to drug delivery. Topical administration may reduce systemic exposure, but its effectiveness is limited by the epidermal barrier and poor penetration into dense fibrotic tissue (156, 157). Systemic administration, while more applicable to diffuse disease, raises concerns about tissue specificity, off-target effects, and long-term safety. Therefore, successful translation will require not only antifibrotic efficacy, but also delivery strategies tailored to lesion extent, disease stage, and the structural features of fibrotic skin. Equally important is the challenge of inhibiting pathological EMT while preserving the reparative functions of epithelial plasticity during normal wound healing. Available evidence suggests that keratinocytes can transiently adopt EMT-like features during wound closure to support migration and re-epithelialization (158), whereas in chronic inflammatory and fibrotic settings, similar programs may become persistent and maladaptive, ultimately promoting profibrotic remodeling. Clinically, keloids often emerge or worsen during periods of hormonal fluctuation, including puberty and pregnancy, suggesting a possible contribution of sex hormones to aberrant scar formation (159, 160). The pronounced female predominance of SSc further supports the relevance of sex-related factors in cutaneous fibrosis (161). Yet, sex-stratified analyses are rarely incorporated into EMT studies, leaving it unclear whether EMT-related programs in fibrotic skin differ according to sex or hormonal status. Future clinical studies should therefore treat sex as an important stratification variable and consider reproductive age, pregnancy-related hormonal context, and postmenopausal status when interpreting therapeutic responses. This may help refine the precision and safety evaluation of EMT-targeted therapies. Clinical translation of EMT-targeted strategies in cutaneous fibrosis is summarized in **Table 3**.

**Table 3:** Clinical translation of EMT-targeted strategies in cutaneous fibrosis.

Intervention measures	Registration number	Disease	State	Result
IL-13 neutralizing antibody	NCT02494206	lymphedema	Completed	This study explored the therapeutic effect of TGF-β and IL-13 neutralizing antibody in secondary lymphedema, especially to reduce the expression of EMT and fibrosis-related genes through TGF-β inhibition. Patients diagnosed with unilateral stage I or stage II breast cancer-related lymphedema (BCRL) received QBX258 treatment (12).
Hyperbaric oxygen therapy (HBOT)	\	Keloids	Completed	The reversal effect of HBOT on the EMT phenomenon in keloids was studied, especially by reducing the expression of EMT markers through HBOT, and improving the blood supply of scar tissue (85).

## Conclusion

Growing evidence supports the involvement of EMT in the initiation and progression of cutaneous fibrosis. However, most existing studies infer that EMT is involved primarily on the basis of changes in EMT-associated molecular markers or signalling pathways. However, systematic mechanistic insight into the precise functional contribution of EMT, its hierarchical regulation, and its dynamic role during inflammation-driven fibrotic progression remains limited. At the same time, current therapeutic methods targeting EMT are mostly confined to preclinical studies, and the translation of these methods into clinical practice faces difficulties. These difficulties include the lack of solid clinical evidence, the need for sufficiently powered and well-designed randomized trials to assess both effectiveness and long-term safety, and technical barriers related to local drug delivery efficiency, target specificity, and large-scale production costs. Although ncRNAs have been comprehensively studied in cancer biology, their roles in regulating EMT in the context of cutaneous fibrosis remain insufficiently understood. Besides ncRNAs, other epigenetic factors, such as DNA methylation and histone modification, also have functions. However, these functions in fibrotic skin diseases remain poorly defined. Therefore, addressing these knowledge gaps will be essential for achieving a more comprehensive understanding of EMT-associated skin fibrosis and for optimizing mechanism-based therapeutic strategies. Cellular senescence is a stress-induced state of relatively stable proliferative arrest, often accompanied by acquisition of the senescence-associated secretory phenotype (SASP) (162), Through the paracrine release of inflammatory cytokines, growth factors, chemokines, and matrix-remodeling mediators, SASP can induce EMT-like changes in neighboring epithelial cells, suggesting a potential link between cellular senescence and epithelial plasticity (163). Cellular senescence has also been implicated in the development and progression of various fibrotic diseases (164). However, the existing evidence is largely derived from distinct disease settings or experimental systems, and direct studies establishing a continuous causal relationship among EMT, cellular senescence, and fibrotic outcomes in cutaneous fibrosis remain limited. Thus, senescence may represent a potential regulator of EMT-like epithelial plasticity in skin fibrosis, but its precise mechanistic role and pathological contribution require further investigation.

Traditionally, the TGF-β signaling pathway has been regarded as the main and standard initiator of EMT in cutaneous fibrosis. However, with the rapid progress of multiomics technologies, the conceptual framework has developed from a TGF-β-centered model into a more complex regulatory network that combines multiple signaling pathways. A growing amount of evidence demonstrates that besides the traditional TGF-β axis, noncanonical signalling pathways such as Wnt, Notch, PI3K/Akt, and MAPK/ERK pathways are necessary in coordinating EMT programs (23). Therefore, this transition from single-target models to multi-dimensional regulatory frameworks not only enriches our comprehension of the molecular mechanisms under skin fibrosis but also draws up a complete pharmacological map that includes wide signaling crosstalk. In this context, selective inhibitors targeting key nodes within EMT-associated pathways have attracted increasing attention in preclinical research. Thus, this provides a systematic theoretical basis for identifying EMT-targeted anti-fibrotic agents that have high translation potential, stronger specificity, and fewer side effects. At the same time, progress in biomaterials science has emphasized the rising potential of interdisciplinary methods that combine functionalized tissue-engineered scaffolds with EMT-targeted pharmacological treatments. By copying physiological microenvironments, bioengineered scaffolds can possibly realize coordinated regulation of EMT signaling networks, hence this helps to overcome the limitations of conventional drug delivery and offers new possibilities for promoting functional skin regeneration and reversing fibrosis. As an emerging therapeutic method, EMT-targeted interventions have the potential to fundamentally change the prevention and treatment of skin fibrotic disorders, with the potential to improve patient outcomes and quality of life.

Notably, although EMT promotes the initiation and progression of fibrosis under chronic inflammatory conditions, it is also an essential physiological mechanism required for effective tissue repair during normal wound healing. Therefore, a critical challenge for future research and therapeutic development lies in achieving a precise balance between suppressing pathological EMT and preserving its indispensable reparative functions. Nevertheless, several key issues remain unresolved. Future studies should clarify whether EMT in cutaneous fibrosis reflects complete transdifferentiation, partial EMT, or a broader form of epithelial plasticity, and define its quantitative contribution relative to resident fibroblasts, pericytes, and other mesenchymal cell populations. Moreover, direct studies linking blood and lymphatic vascular dysfunction to EMT-like epithelial plasticity in cutaneous fibrosis remain scarce and warrant further investigation. Resolving these questions will require the integration of single-cell and spatial multi-omics, lineage-tracing strategies in appropriate experimental models, and advanced bioengineered skin systems that more faithfully recapitulate the fibrotic microenvironment. Such approaches may help identify disease stage-specific EMT programs, uncover clinically relevant biomarkers, and support the development of selective interventions that suppress pathological EMT without compromising physiological wound repair. Ultimately, the therapeutic value of EMT-directed strategies in cutaneous fibrosis will depend not on broad EMT suppression, but on the precise modulation of disease-relevant fibrotic networks.
